# Supplementary material for: Within- and cross-species predictions of plant specialized metabolism genes using transfer learning
Source: In Silico Plants. 2020 Jul 30;2(1):diaa005. doi: 10.1093/insilicoplants/diaa005 (PMC7731531; doi:10.1093/insilicoplants/diaa005)
Supplement: diaa005_suppl_Supplementary_Figure_S1 [file diaa005_suppl_supplementary_figure_s1.pdf]

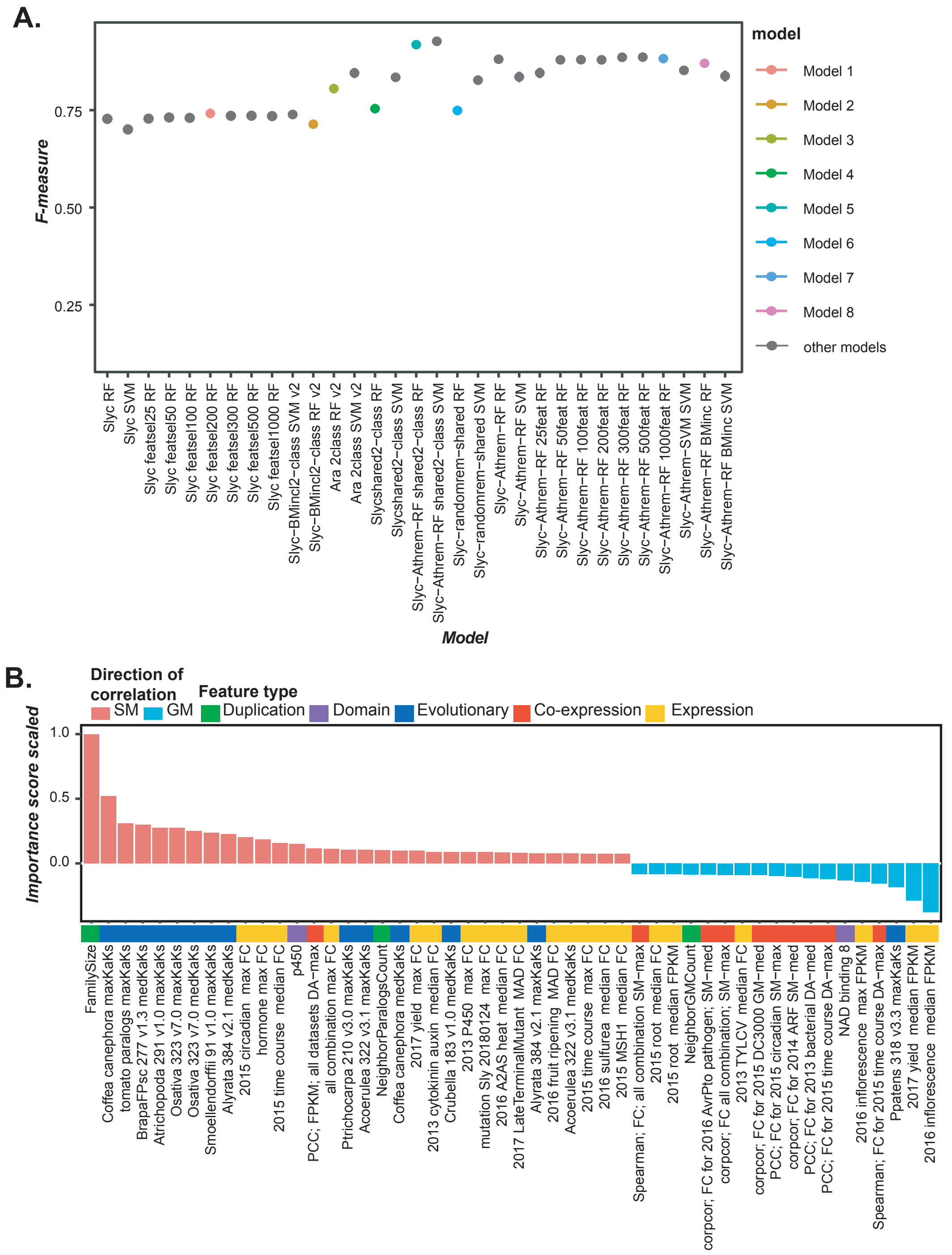

Supplemental Figure 1: Comparison of all model scores and feature importance values for Model 1 (A) Comparison of model scores. F-measure is shown on the y-axis and model is shown on the x-axis. Model type is denoted by color. Gray indicates Models 1-8 variants (i.e., different ML algorithms and/or numbers of features used) that are not described in the text. RF: Random Forest. SVM: Support Vector Machine. featsel25-1000: features selected, sets of 25 to 1000. For model names, see Table S2. (B) Bar plot of the top 50 most important features for Model 1. The importance score is on the y-axis and all scores are normalized to the score of the most important feature, which was set as 1. Red bars represent features that are enriched for SM genes while the blue bars represent features enriched for GM genes. Features are listed along the x-axis, with the color denoting the feature category.
