# Supplementary material for: Within- and cross-species predictions of plant specialized metabolism genes using transfer learning
Source: In Silico Plants. 2020 Jul 30;2(1):diaa005. doi: 10.1093/insilicoplants/diaa005 (PMC7731531; doi:10.1093/insilicoplants/diaa005)
Supplement: diaa005_suppl_Supplementary_Figure_S2 [file diaa005_suppl_supplementary_figure_s2.pdf]

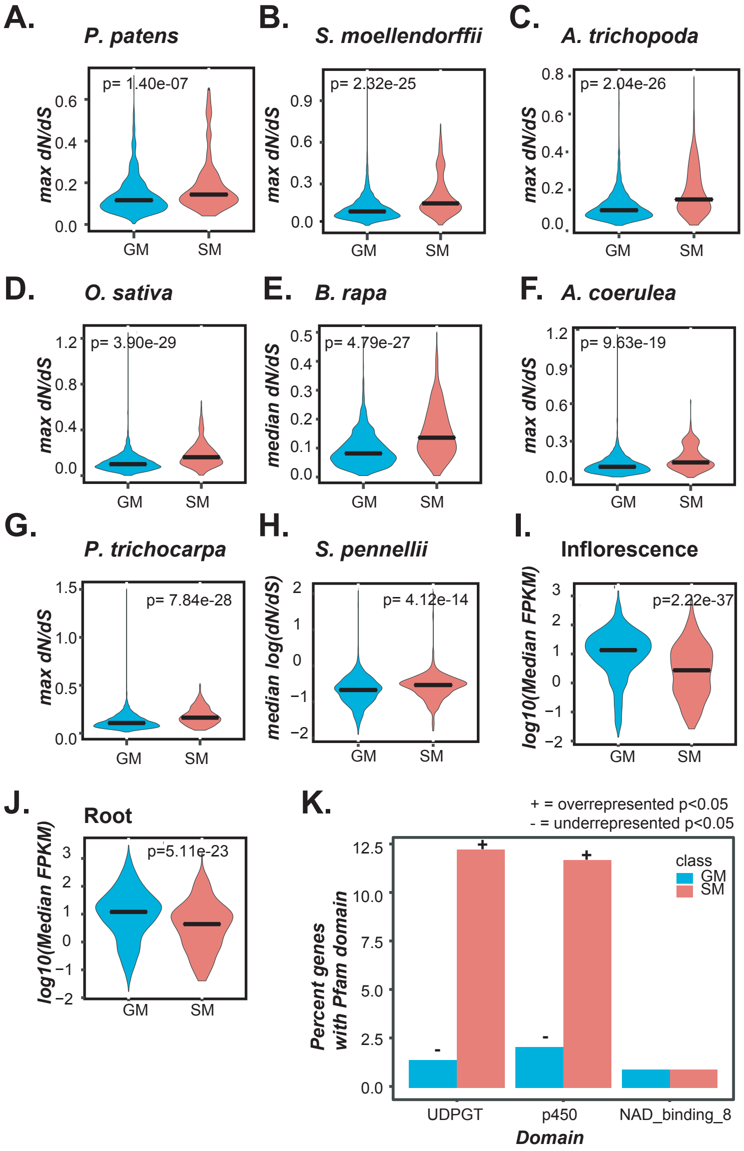

Supplemental Figure 2: Important features for Model 1.
(A-K) Distributions or bar plots of feature values for TomatoCyc-annotated SM and GM genes. (A-J) Significance determined by the Mann-Whitney U test. (A-H) Distributions of the maximum or median dN/dS value for a given gene relative to their homolog in *P. patens, S. moellendorffii, A. trichopoda, O. sativa, B. rapa, A. coerulea, P. trichocarpa* and *S. pennellii*. (I, J) Distributions of log 10 of median FPKM values for the Inflorescence data set and Root data set. (K) Percent of genes with a given Pfam domain. Overrepresentation (+) and underrepresentation (-) was determined using those genes with a p-value less than 0.05 from a Fisher's Exact test between SM and GM genes with Benjamin-Hochberg multiple testing correction.
