# Supplementary material for: Within- and cross-species predictions of plant specialized metabolism genes using transfer learning
Source: In Silico Plants. 2020 Jul 30;2(1):diaa005. doi: 10.1093/insilicoplants/diaa005 (PMC7731531; doi:10.1093/insilicoplants/diaa005)
Supplement: diaa005_suppl_Supplementary_Figure_S3 [file diaa005_suppl_supplementary_figure_s3.pdf]

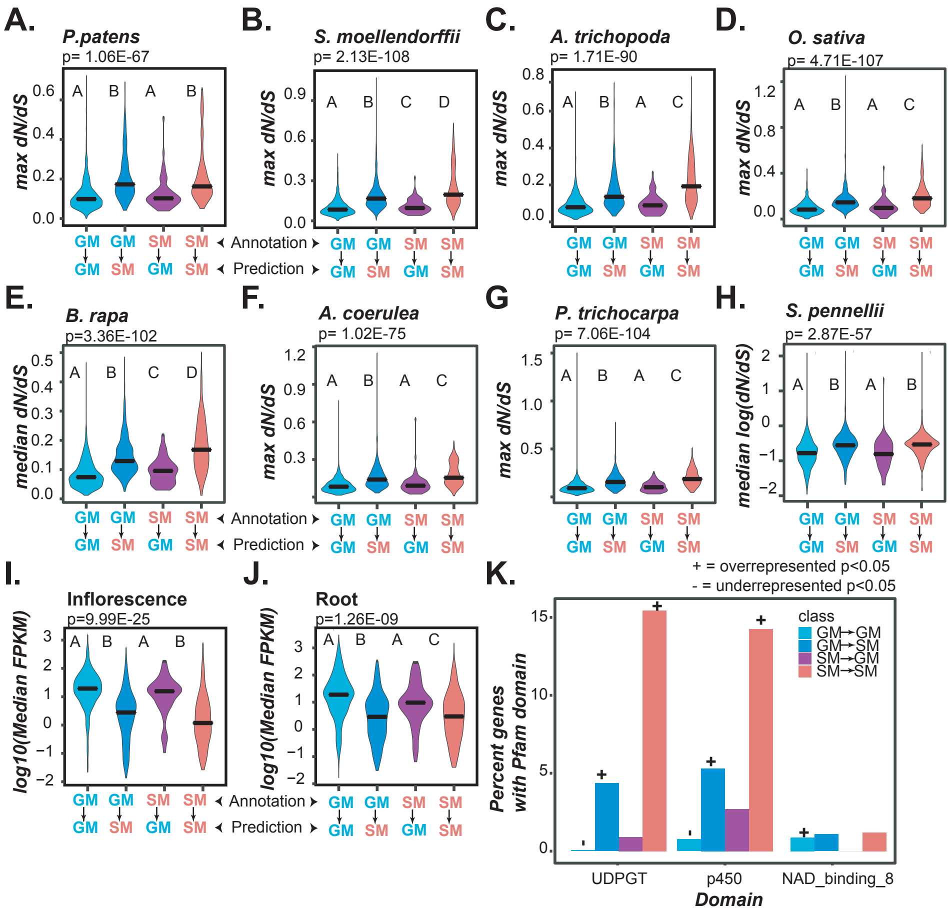

Supplemental Figure 3: Features important for SM vs. GM predictions

For all distributions of each predicted class, GM›GM represents GM genes predicted by Model 1 as GM, GM SM represents GM genes predicted by Model 1 as SM, SM›GM represents SM genes predicted by Model 1 as GM, and SM›SM represents SM genes predicted by Model 1 as SM. Significant differences between continuous variables were determined by the Kruskal-Wallis test (A-J) and post-hoc comparisons were made using Dunn's test. Different letters indicate statistically significant differences between groups ($P < 0.05$). For binary data (K), overrepresentation (+) and underrepresentation (-) were determined by the Fisher's Exact test where (+) is significant overrepresentation of a predicted class and (-) is significant underrepresentation. A p-value < 0.05 after Benjamin-Hochberg multiple testing correction was considered significant. (A-H) Distributions of the maximum or median dN/dS value for a given gene from comparisons to its homolog in *P. patens, S. moellendorffii, A. trichopoda, O. sativa, B. rapa, A. coerulea, P. trichocarpa* and *S. pennellii*. (I, J) Distributions of log10 (median FPKM) values for the Inflorescence (I) and Root (J) data sets. (K) Percentage of genes with a given Pfam domain.
