# Supplementary material for: Within- and cross-species predictions of plant specialized metabolism genes using transfer learning
Source: In Silico Plants. 2020 Jul 30;2(1):diaa005. doi: 10.1093/insilicoplants/diaa005 (PMC7731531; doi:10.1093/insilicoplants/diaa005)
Supplement: diaa005_suppl_Supplementary_Figure_S4 [file diaa005_suppl_supplementary_figure_s4.pdf]

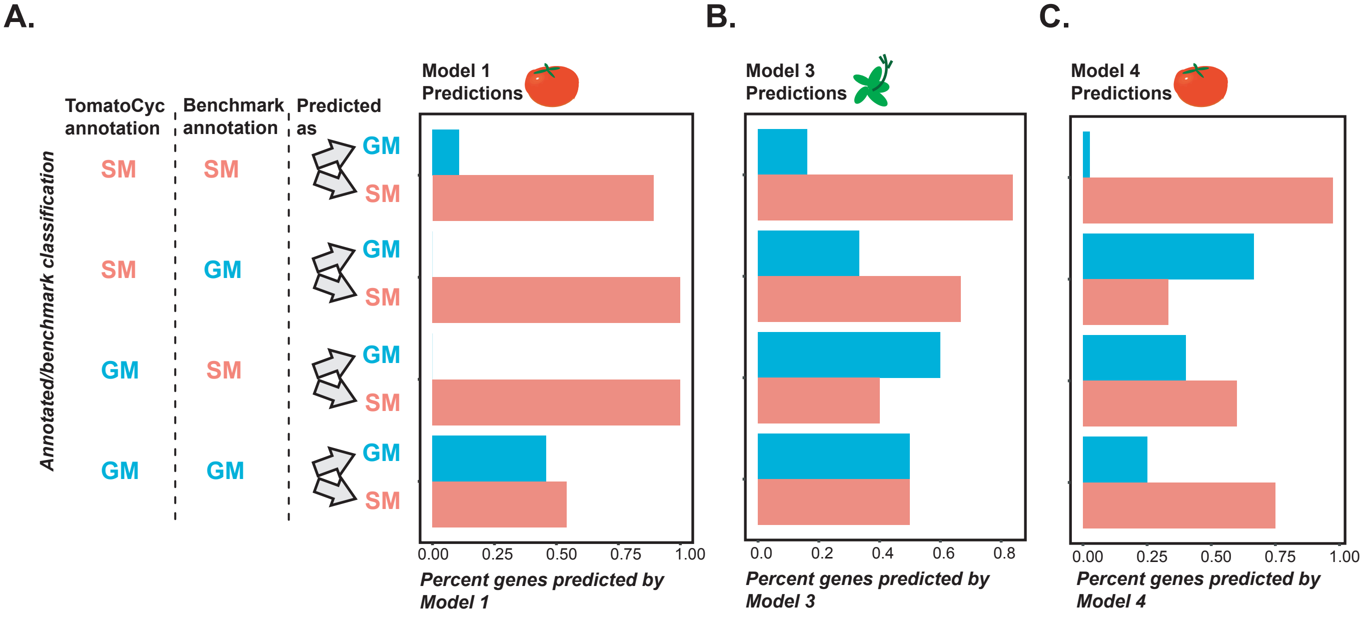

Supplemental Figure 4: Predictions of manually annotated genes
(A-C) Bar plots showing the percentage of manually annotated benchmark genes predicted as SM or GM. The original annotation from TomatoCyc is shown first, followed by the benchmark annotation and then the prediction. (A) Predictions for Model 1, (B) Predictions for Model 3, (C) Predictions for Model 4.
