# Supplementary material for: Within- and cross-species predictions of plant specialized metabolism genes using transfer learning
Source: In Silico Plants. 2020 Jul 30;2(1):diaa005. doi: 10.1093/insilicoplants/diaa005 (PMC7731531; doi:10.1093/insilicoplants/diaa005)
Supplement: diaa005_suppl_Supplementary_Figure_S5 [file diaa005_suppl_supplementary_figure_s5.pdf]

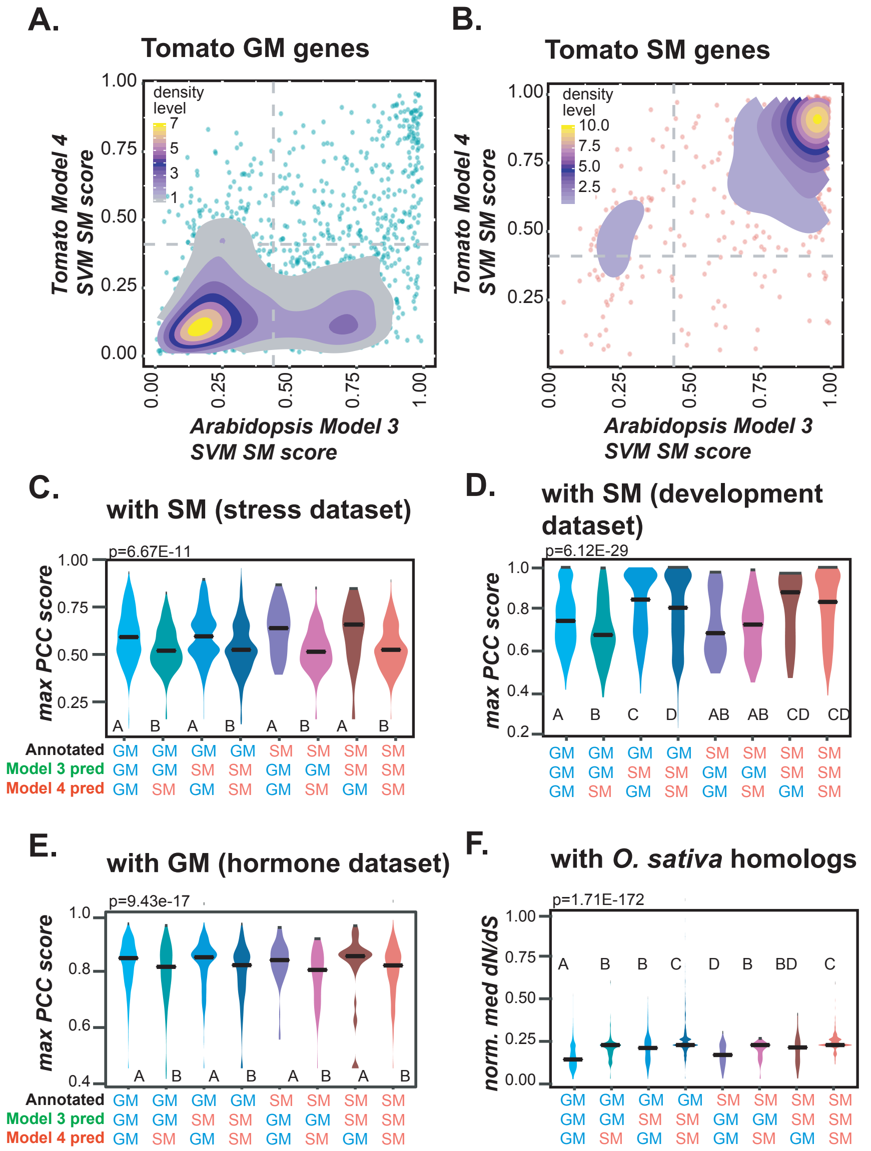

Supplemental Figure 5: S. lycopersicum and A. thaliana model comparison and model performance (A-B) Comparison of the SM score distributions for tomato Model 4 (y-axis) and Arabidopsis Model 3 (x-axis). Support Vector Machine (SVM) and a shared feature set were used for both models. Density of data points ranges from high (yellow) to medium (blue-purple) to low (white). (A) SM scores for GM genes; (B) SM scores for SM genes; (C-F) Feature distributions for annotated SM and GM genes that are predicted as SM or GM genes by Arabidopsis Model 3 and tomato Model 4. The x-axis lists the annotations for each group of genes predicted using Arabidopsis Model 3 and tomato Model 4. P-values are from the Kruskal-Wallis test and post-hoc comparisons were made using the Dunn's test. Different letters indicate statistically significant differences between groups ($P < 0.05$). (C) maximum Pearson's Correlation Coefficient (PCC) between a given gene and all other SM genes under stress conditions; (D) maximum PCC between a given gene and all other SM genes during development; (E) maximum PCC between a given gene and all other GM genes under hormone treatment; (F) normalized median dN/dS values between tomato or Arabidopsis genes and their homologs in *O. sativa*.
