# Supplementary material for: Within- and cross-species predictions of plant specialized metabolism genes using transfer learning
Source: In Silico Plants. 2020 Jul 30;2(1):diaa005. doi: 10.1093/insilicoplants/diaa005 (PMC7731531; doi:10.1093/insilicoplants/diaa005)
Supplement: diaa005_suppl_Supplementary_Figure_S6 [file diaa005_suppl_supplementary_figure_s6.pdf]

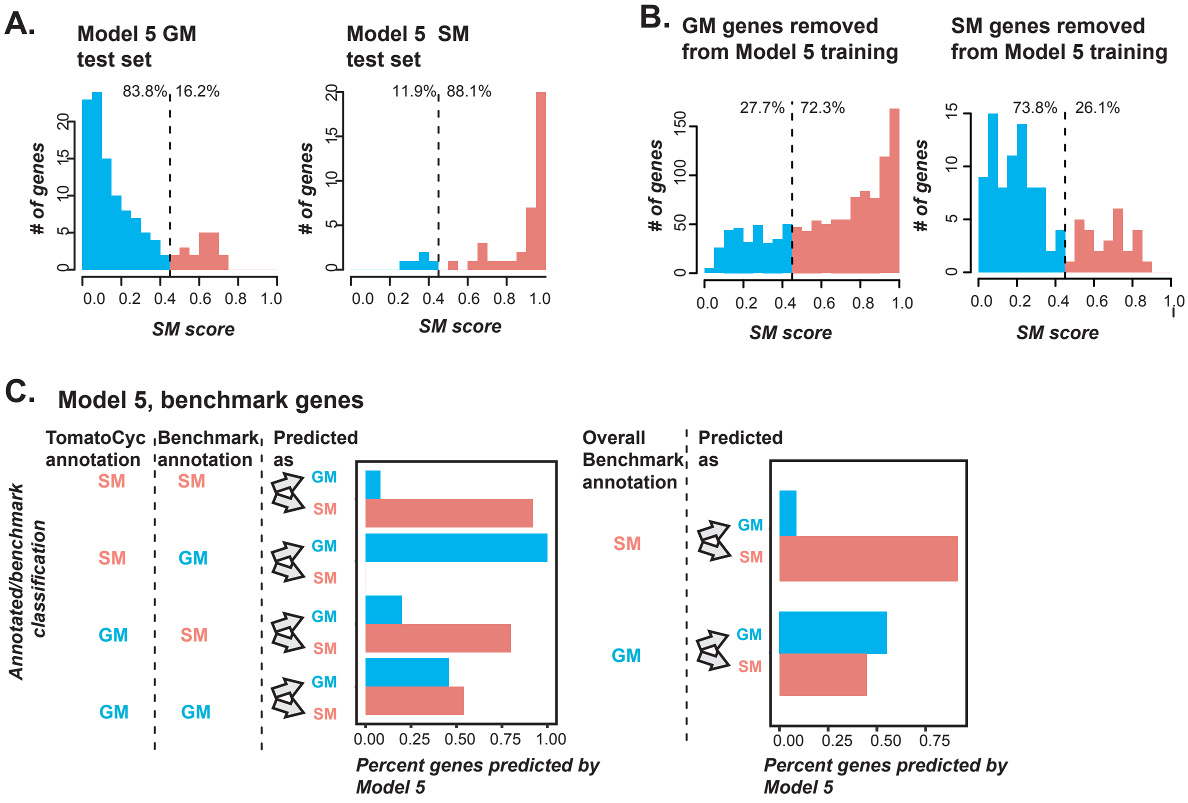

Supplemental Figure 6: Benchmark and test set predictions from finalized models with *A. thaliana* mis-predictions removed (Models 5)

Plots A and B show distributions of SM likelihood scores. (A) Model 5 test set SM and GM genes, which were held out from the model building process completely. (B) TomatoCyc SM and GM genes with annotations opposite to Arabidopsis Model 3 predictions removed from the filtered training set. For plots (A, B): SM likelihood score is shown on the x-axis, number of genes is on the y-axis. Prediction threshold, based on the score with the highest F-measure, is indicated by the dotted line, and predicted SM genes are shown to the right of the line in red while predicted GM genes are shown to the left of the line in blue. (C) Bar plots showing the percentage of manually annotated benchmark genes predicted as SM or GM by Model 5. For the first barplot, the original annotation from TomatoCyc is shown first, followed by the benchmark annotation and then the prediction. The second barplot shows overall benchmark predictions (not divided by TomatoCyc annotations).
