# Supplementary material for: Within- and cross-species predictions of plant specialized metabolism genes using transfer learning
Source: In Silico Plants. 2020 Jul 30;2(1):diaa005. doi: 10.1093/insilicoplants/diaa005 (PMC7731531; doi:10.1093/insilicoplants/diaa005)
Supplement: diaa005_suppl_Supplementary_Figure_S7 [file diaa005_suppl_supplementary_figure_s7.pdf]

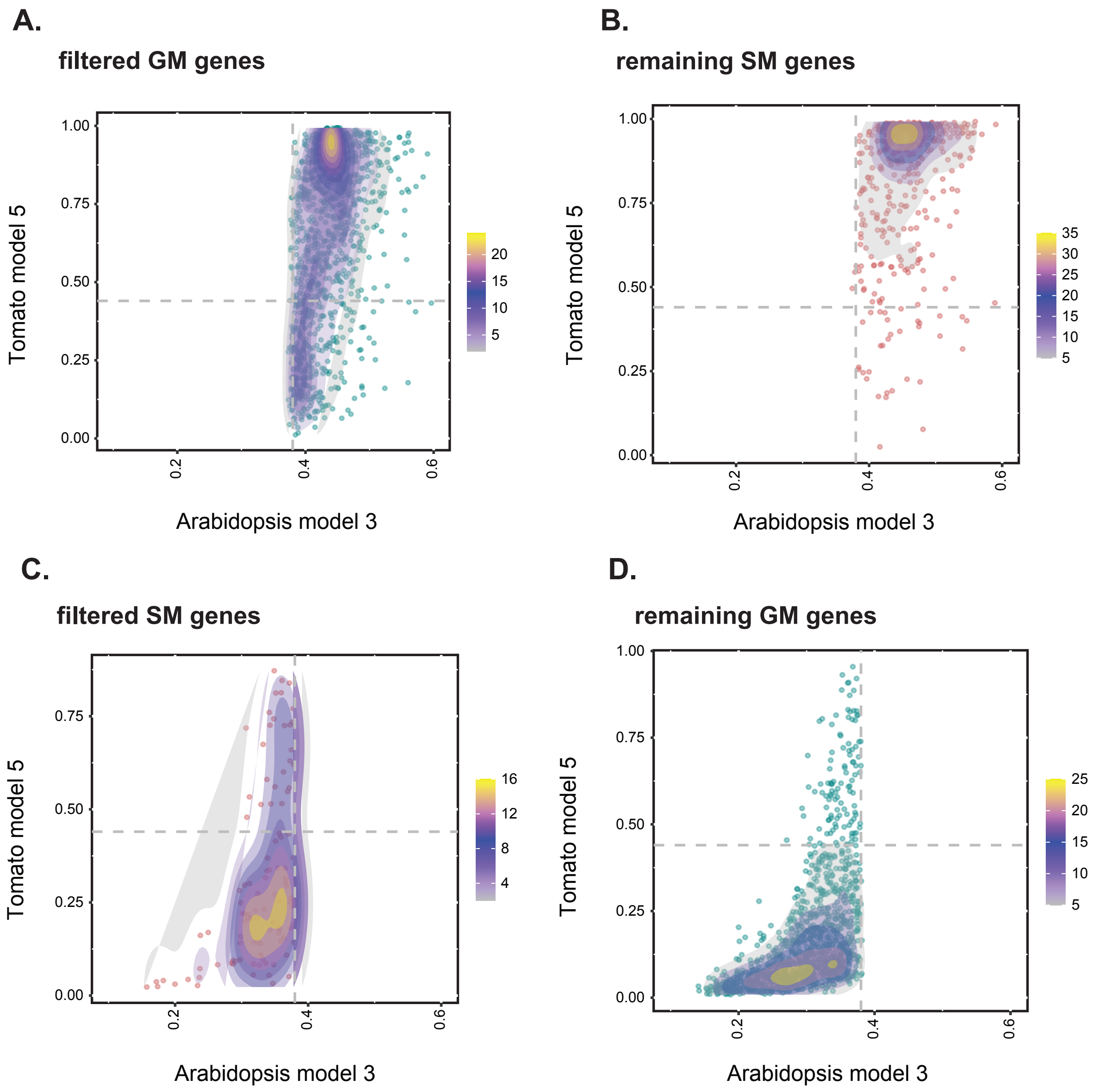

Supplemental Figure 7: Arabidopsis model 3 and Tomato model 5 comparison
Plots A-D show gene scores from Arabidopsis model 3 on the x-axis and Tomato model 5 on the y-axis. Color: data point density ranges from high (yellow) to medium (purple), to low (fading purple). (A) filtered GM genes- removed from model 5 training (B) remaining SM genes- kept in model 5 training (C) filtered SM genes- removed from model 5 training (D) remaining GM genes- kept in model 5 training.
