# Supplementary material for: Within- and cross-species predictions of plant specialized metabolism genes using transfer learning
Source: In Silico Plants. 2020 Jul 30;2(1):diaa005. doi: 10.1093/insilicoplants/diaa005 (PMC7731531; doi:10.1093/insilicoplants/diaa005)
Supplement: diaa005_suppl_Supplementary_Figure_S8 [file diaa005_suppl_supplementary_figure_s8.pdf]

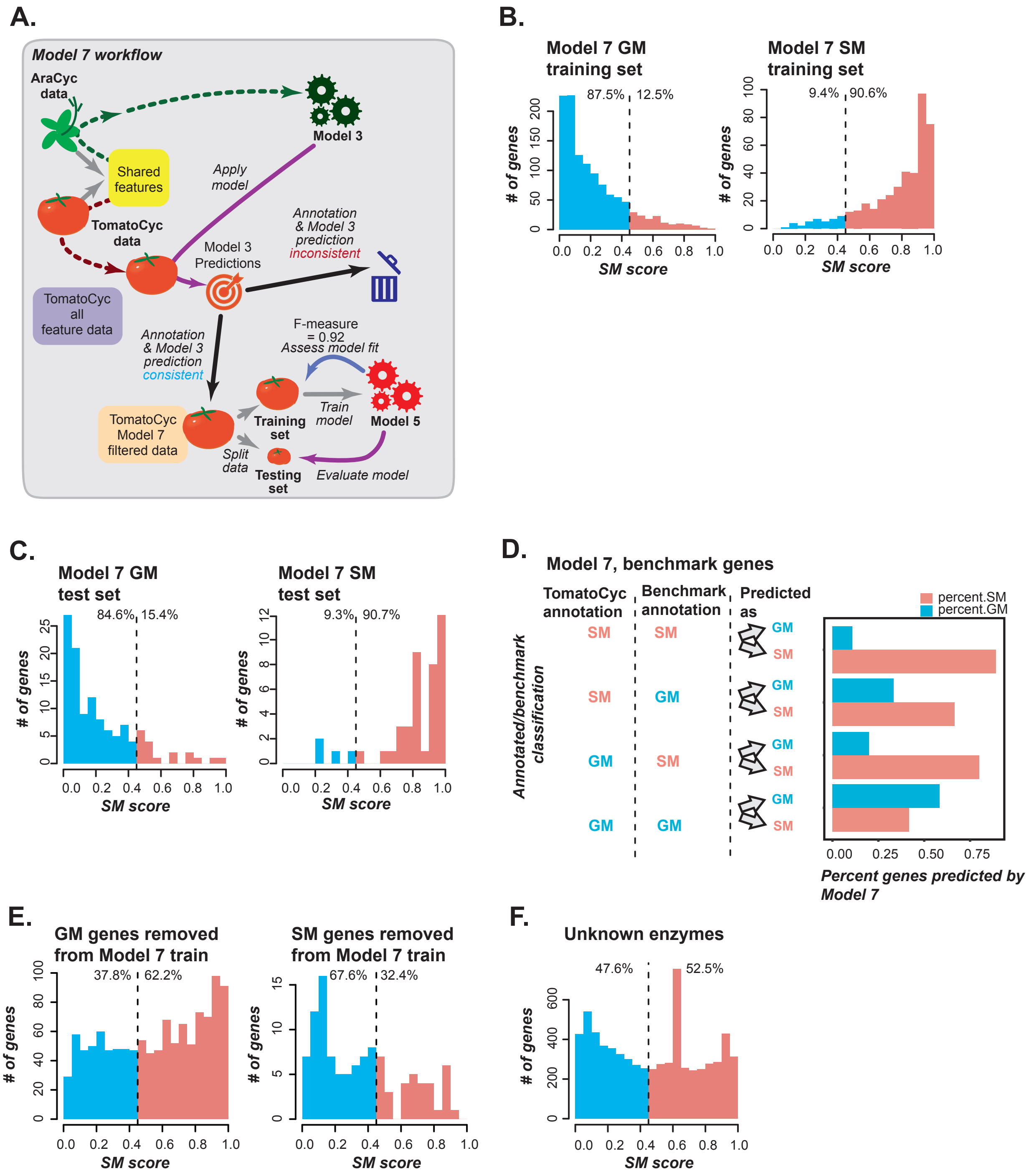

Supplemental Figure 8: Benchmark and test set predictions from finalized models with *A. thaliana* mis-predictions removed (Models 7)

(A) Schematic diagram showing the application of tomato Model 7 to tomato. The full tomato feature dataset was used to build a binary model using TomatoCyc SM and GM annotations after removing genes mis-predicted by Arabidopsis Model 3. The model was then applied to tomato genes. (B) TomatoCyc filtered training set SM and GM genes from tomato Model 7. (C) Model 7 test set: SM and GM genes, which were held out completely from the tomato Model 7 building process. (D) Bar plot showing the percentage of manually annotated benchmark genes predicted as SM or GM by Model 7. The original annotation from TomatoCyc is shown first, followed by the benchmark annotation and then the prediction. (E) SM and GM genes removed from Model 7 training set (F) Unannotated tomato enzymes. For plots (B, C and E, F): SM likelihood score is shown on the x-axis, number of genes is on the y-axis. Prediction threshold, based on the score with the highest F-measure, is indicated by the dotted line, and predicted SM genes are shown to the right of the line in red while predicted GM genes are shown to the left of the line in blue.
