# Supplementary material for: Within- and cross-species predictions of plant specialized metabolism genes using transfer learning
Source: In Silico Plants. 2020 Jul 30;2(1):diaa005. doi: 10.1093/insilicoplants/diaa005 (PMC7731531; doi:10.1093/insilicoplants/diaa005)
Supplement: diaa005_suppl_Supplementary_Figure_S9 [file diaa005_suppl_supplementary_figure_s9.pdf]

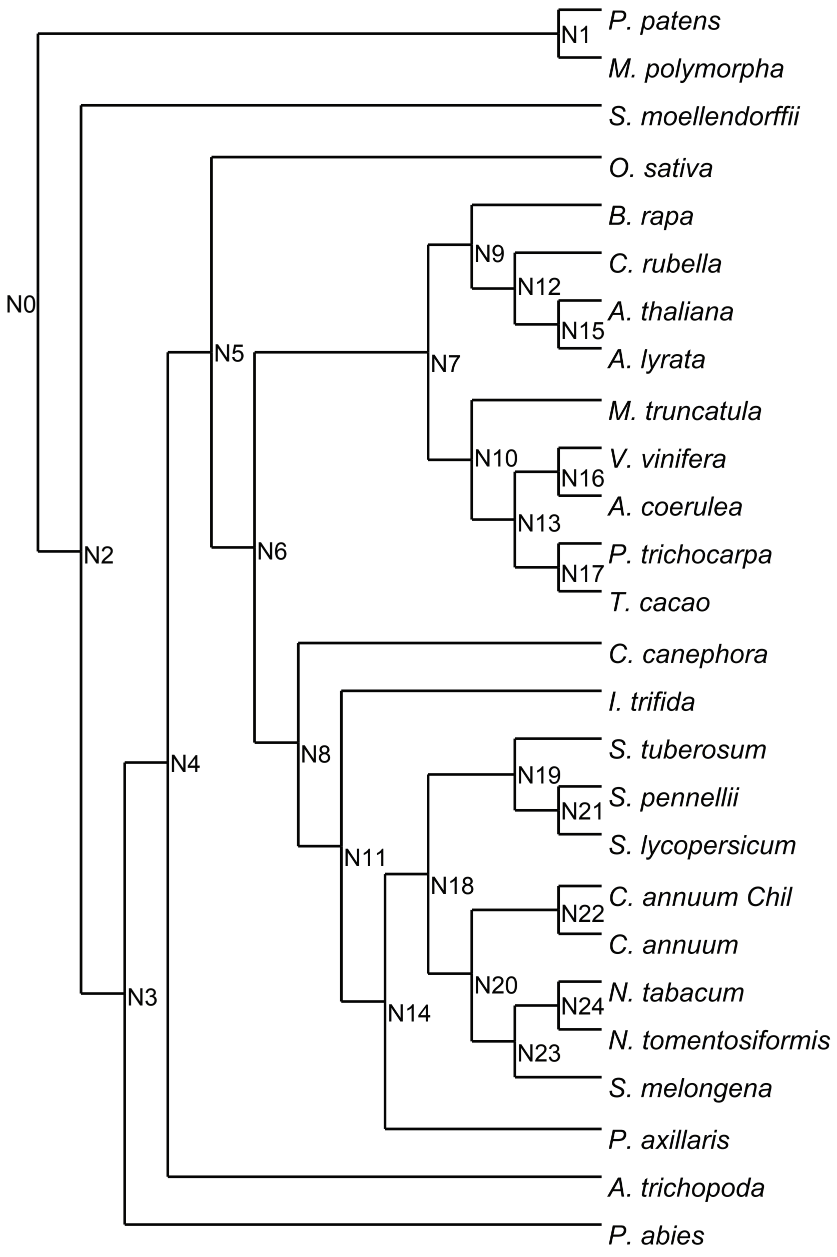

Supplemental Figure 9: Speciation nodes
Phylogenetic tree of 26 species showing speciation nodes (N0-N24). Most recent gene duplication node in text refers to the speciation node where gene was last duplicated.
